# A Thymus-Independent Artificial Organoid System Supports Complete Thymopoiesis from Rhesus Macaque-Derived Hematopoietic Stem and Progenitor Cells

**DOI:** 10.3390/biomedicines13112692

**Published:** 2025-11-01

**Authors:** Callie Wilde, Saleem Anwar, Yu-Tim Yau, Sunil Badve, Yesim Gökmen-Polar, John D. Roback, Rama Rao Amara, R. Paul Johnson, Sheikh Abdul Rahman

**Affiliations:** 1 Department of Pathology and Laboratory Medicine, Emory University School of Medicine, Atlanta, GA 30322, USA; 2 Division of Microbiology and Immunology, Emory Vaccine Center, Emory National Primate Research Center, Emory University, Atlanta, GA 30329, USA; 3 Department of Microbiology and Immunology, Emory University School of Medicine, Emory University, Atlanta, GA 30322, USA; 4 Infectious Disease Division, Department of Medicine, Emory University School of Medicine, Atlanta, GA 30322, USA

**Keywords:** T cell regenerative immunology, Rhesus-thymus independent artificial organoid, nonhuman primate model, hematopoietic stem and progenitor cells, T cell lineage differentiation, Ex-vivo T cell receptor (TCR) selection

## Abstract

**Background:** T cell regeneration in the thymus is intrinsically linked to the T cell-biased lineage differentiation of hematopoietic stem and progenitor cells (HSPCs). Although nonhuman primates (NHPs) serve as indispensable models for studying thymic output under physiological and pathological conditions, a non-animal technology facilitating efficient TCR-selected T cell development and evaluating T cell output from NHP-derived HSPCs has been lacking. To address this gap, we established a rhesus macaque-specific artificial thymic organoid (RhATO) modeling primary thymus-tissue-free thymopoiesis. **Methods:** The RhATO was developed by expressing Rhesus macaque (RM) Delta-like Notch ligand 1 in mouse bone marrow stromal cell line (MS5-RhDLL1). The bone marrow-derived HSPCs were aggregated with MS5-RhDLL1 and cultured forming 3D artificial thymic organoids. These organoids were maintained under defined cytokine conditions to support complete T cell developmental ontogeny. T cell developmental progression was assessed by flow cytometry, and TCR-selected subsets were analyzed for phenotypic and functional properties. **Results:** RhATOs recapitulated the complete spectrum of thymopoietic events, including emergence of thymus-seeding progenitors, CD4^+^CD3^−^ immature single-positive and CD4^+^CD8^+^ double-positive early thymocytes, and mature CD4^+^ or CD8^+^ single-positive subsets. These subsets expressed CD38, consistent with the recent thymic emigrant phenotype, and closely mirrored canonical T cell ontogeny described in humans. RhATO-derived T cells were TCR-selected and demonstrated cytokine expression upon stimulation. **Conclusions:** This study provides the first demonstration of an NHP-specific artificial thymic technology that faithfully models thymopoiesis. RhATO represents a versatile ex vivo platform for studying T cell development, immunopathogenesis, and generating TCR selected T cells.

## 1. Introduction

T cell development involves a stepwise transformation of hematopoietic stem and progenitor cells (HSPCs), which undergo an initial T lineage commitment in the bone marrow to generate thymus-seeding progenitors (TSPs) [1,2]. Upon thymic entry, TSPs—typically CD4^−^CD8^−^ double negative CD34^+^CD38^−/+^CD7^−/+^ population—upregulate markers such as CD7 and the T cell factor 1 (TCF-1), differentiating into immature single-positive (ISP) thymocytes [2,3,4,5,6]. ISPs express CD4 but lack CD3, and transition into CD4^+^CD8^+^ double-positive (DP) thymocytes, which subsequently upregulate CD3. Positive selection via MHC-I or MHC-II leads to the emergence of CD8^+^ and CD4^+^ single-positive (SP) T cells, respectively. The SP CD8 and CD4 T cells leave the thymus and enter circulation, where they are called recent thymic emigrants and express high levels of surface CD38 [7]. Accurately modeling this ontogeny ex vivo is critical not only for generating therapeutic T cells but also for advancing our understanding of T cell regenerative biology and thymic disruption in disease.

Nonhuman primates (NHPs) offer a physiologically relevant preclinical model for studying T cell development in the context of chronic and infectious diseases [8,9]. Prior attempts to model thymopoiesis from NHP-derived HSPCs demonstrated T cell selection and development [10,11,12]. These systems required co-culturing HSPCs with either rhesus- or porcine-derived primary thymus stromal cells. Similar strategies in human systems employed CD34^+^ HSPCs with thymic epithelial components [11,12,13,14,15,16]; however, broader adoption has been limited by the need for primary thymic tissue and its short shelf life. To overcome these limitations, murine stromal cell lines (OP9 or MS-5) were engineered to express Notch ligands DLL1 or DLL4, enabling thymus-independent co-culture systems [6,17,18,19,20]. When co-cultured with HSPCs from human, NHP, or mouse origins, these systems biased differentiation toward the T cell lineage, in contrast to the default B or NK pathways [18,21,22,23] underscoring the significance of Notch signaling.

Despite exhibiting a conserved Notch pathway, human or NHP HSPCs cultured with the OP9/MS5-DLL1/4 system have shown inefficient selection of T cell precursors leading to fewer productively TCR rearranged T cell subset development [6,18,21,23,24,25,26]. Improved outcomes were observed when human or mouse HSPCs were aggregated with stromal cells expressing species-specific DLL1 in 3D culture [27,28]. These so-called artificial thymic organoid systems generated consistent T cell developmental outcomes with enhanced TCR^+^ T cell selection. Nonetheless, studies testing ex vivo T cell generation methods in a NHP model is limiting [11,21]. The field lacks a validated, species-specific 3D organoid system for NHPs capable of supporting functional T cell output.

Here, we report the development and validation of a Rhesus macaque-specific artificial thymic organoid (RhATO) system that supports efficient ex vivo thymopoiesis from CD34^+^CD3^−^ bone marrow-derived HSPCs. Using MS5 stromal cells engineered to express RM-specific DLL1 and aggregated in 3D cultures with RM-derived progenitors, the RhATO platform recapitulated sequential thymic intermediates—including CD7^+^ TSPs, CD4^+^CD3^−^ ISPs, CD4^+^CD8^+^ DPs—and yielded mature CD3^+^TCR^+^ SP CD4^+^ and CD8^+^ T cell subsets expressing CD38, resembling recent thymic emigrants. Notably, the system also supported T cell differentiation from human HSPCs. This study establishes an RhATO as the first NHP-specific, artificial organoid system capable of supporting a primary thymus-tissue-free T cell development.

## 2. Materials and Methods

### 2.1. Construction of Recombinant Mouse Bone Marrow Stromal Cell (MS-5) Expressing Rhesus macaque (Macaca mulatta) Delta-Like Canonical Notch Ligand 1 (DLL1)

Construct design: HIV based lentiviral (LV) expression cassette was designed to express coding sequence of *Macaca mulatta* (Rhesus macaque) Delta-like canonical Notch ligand 1 (DLL1) based on reported sequence (NCBI RefSeq: XP_014993255.1). Briefly, 5′ long terminal repeat (LTR) was placed under rouse sarcoma virus (RSV) promoter replacing proviral promoter U3 region (ΔU3). Rh-DLL1 was codon optimized for expression in murine system and was placed under MNDU3 promoter. To enable fluorescent tracking, enhanced green fluorescent protein (eGFP) was co-expressed via internal ribosome entry site (IRES). For antibiotic selection, puromycin *N*-acetyltransferase coding sequence was incorporated downstream of mPGK promoter. Vector construction and generation of VSV-G pseudotyped particles were outsourced to VectorBuilder Inc., Chicago, IL, USA.

Lentiviral transduction of MS-5 cell line: Murine bone marrow stromal MS-5 cell line (Creative Bioarray, Shirley, NY, USA., Cat #CSC-C2763) were maintained in complete DMEM supplemented with 10% fetal bovine serum (FBS; Cat #A5256701, Thermo Fisher scientific, Asheville, NC, USA), 1% l-glutamine, 1% HEPES buffer, and 1% penicillin–streptomycin at 37 °C in 5% CO_2_. For transduction, 80% confluent MS-5 cultures were seeded at 1 × 10^6^ cells/well in six-well plates (Day 0). On Day 1, medium was replaced with 1 mL DMEM containing 5 µg/mL polybrene and LV particles encoding *RhDLL1*. Cells were incubated overnight at 37 °C. On Day 2, viral supernatant was replaced with fresh DMEM, and cells were further incubated for 24 h. On Day 3, selection was initiated using 5 µg/mL puromycin, with medium refreshed every 48–72 h until complete selection was achieved. Recombinant MS-5-RhDLL1 cells were expanded and cryopreserved for downstream assays.

### 2.2. Characterization of Transduced MS-5 Expressing RhDLL1

Validation of expression: The transduced MS5 cells were characterized for eGFP as well as surface expression of RhDLL1. In total, 70–80% confluent MS5 as well as MS5-RhDLL1 cell lines were trypsinized, washed with PBS, and counted. A sample of 1 × 10^6^ cells, each from MS5 and MS5-Rh-DLL1, was transferred into 5mL FACS tubes and centrifuged at 300× *g* for 5 min. The supernatant was discarded and the cell pellet was resuspended in 100 μL FACS buffer containing live/dead stain (CAT #L34975, Invitrogen, Carlsbad, CA, USA) and anti-DLL1 antibody (CAT #564414, BD Biosciences, San Jose, CA, USA). The cell and antibody mixture was incubated on ice for 20 min followed by two washes by FACS buffer by centrifugation at 300× *g* for 5 min. The supernatant was discarded and the stained cell pellet was resuspended in 0.5 mL FACS buffer. The flow cytometry acquisition was performed to detect the expression of eGFP and RhDLL1. MS5 cells were used as negative control.

Mycoplasma control: The RhDLL1 cell line was tested for Mycoplasma contamination using the Universal Mycoplasma Detection Kit (ATCC, Manassas, VA, USA, Cat. No. 30-1012K) following the manufacturer’s protocol. Cells (10^4^–10^5^) were harvested without enzymatic dissociation to preserve Mycoplasma membranes, pelleted by centrifugation (12,000 rpm, 3 min, 4 °C), and lysed in 50 µL lysis buffer at 37 °C for 15 min. A 5 µL aliquot of lysate was added to a 25 µL PCR reaction containing 2× PCR Master Mix, Mycoplasma-specific primers (targeting 16S rRNA), and PCR-grade water. Reactions included positive and negative controls. PCR was run with the following conditions: 94 °C for 2 min; 35 cycles of 94 °C for 30 s, 55 °C for 30 s, 72 °C for 1 min; and a final extension at 72 °C for 10 min. Products were resolved on a 1.5% ethidium bromide-stained agarose gel in 1× TAE buffer, run at 100–120 V for 30–40 min. A 434 bp band indicated a positive result; absence of a band indicated no contamination, with proper control validation.

### 2.3. Bioethical Approvals

Bone marrow aspirates were obtained from the Rhesus macaques through the Emory National Primate Research Center’s Biological Material Procurement program in accordance with the Animal Welfare Act and the National Institutes of Health (NIH) (Bethesda, MD, USA) Guide for the Care and Use of Laboratory Animals, using protocols approved by the Emory University Institutional Animal Care and Use Committee. Biospecimens from the Rhesus macaques were obtained from the study approved under IACUC number IPROTO202400000081, approved on 9 August 2024. Bone marrow aspirates were obtained from young adult Rhesus macaques. The human samples were obtained from discarded mobilized peripheral blood. De-identified and discarded samples were collected in accordance with the Emory Center for Transfusion and Cellular Therapies sample release authorization. The samples were obtained following approval from an institutional ethical procedure under IRB00045821.

### 2.4. Processing of Rhesus macaque Thymus Tissue

The intact thymus tissue was processed to obtain thymocytes. Briefly, the thymus was sliced into small fragments using a sterile scalpel in a 10 cm^2^ round culture dish. The tissue fragments were further minced with sterile scissors. To facilitate thymocyte release, the minced tissue was gently pressed against the culture dish using a sterile syringe plunger, followed by emulsification through repeated passage through an 18-gauge needle to disrupt larger aggregates. The resulting suspension was filtered through a 100 µm cell strainer. The filtrate was washed with PBS by centrifugation at 300× *g* for 5 min. The supernatant was discarded, and the cell pellet was resuspended in 3 mL of ACK lysis buffer (Cat. #118-156-101, Quality Biological Inc., Gaithersburg, MD, USA) and incubated for 3–4 min at room temperature. Following lysis, the mixture was diluted with FBS-supplemented PBS to a final volume of 25 mL. Cells were washed twice with FBS-supplemented PBS, and the final pellet was resuspended in an appropriate volume of buffer, counted, and either used immediately or stored. All steps and buffers were maintained on ice unless otherwise specified.

### 2.5. Processing of Rhesus macaque Peripheral Blood Mononuclear Cells

Blood samples were collected in Vacutainer CPT™ mononuclear cell preparation tubes (Avantor Inc, Suwanee, GA, USA) and centrifuged at 1500× *g* for 30 min. Following centrifugation, the plasma fraction was removed, and the buffy coat was transferred into a sterile 50 mL conical tube. Cells were washed with PBS by centrifugation at 300× *g* for 5 min. The supernatant was discarded, and the pellet was resuspended in 3 mL of ACK lysis buffer and incubated for 3–4 min at room temperature. The cell suspension was then diluted with FBS-supplemented PBS to a final volume of 25 mL and centrifuged at 300× *g* for 5 min. Cells were further washed with FBS-supplemented PBS as before, and the final pellet was resuspended in an appropriate volume of buffer, counted, and either used immediately or stored.

### 2.6. Processing of Human Mobilized Peripheral Mononuclear Cells

Human mobilized peripheral mononuclear cells were obtained from the Emory Hospital Cell and Gene Therapy Unit through established sample release procedure. These samples were left over after the patient’s treatment and were considered as discarded material. The PBMCs were centrifuged at 300× *g* for 5 min. After discarding the supernatant, the cell pellet was treated with ACK lysis buffer (CAT #118-156-101, Quality Biological) to remove RBCs. The cell pellet was resuspended in 3 mL ACK lysis buffer and incubated for 3–4 min at RT. After incubation, the ACK buffer/cell mixture was topped up with FBS-supplemented PBS and adjusted the volume to 25 mL and centrifuged at 300× *g* for 5 min. Cells were washed twice with FBS-supplemented PBS as before, and final pellet was resuspended in appropriate volume of buffer, counted, and used or stored.

### 2.7. Processing of Rhesus macaque Bone Marrow-Derived Biospecimen

Bone marrow aspirates were obtained from young adult Rhesus macaques either through the Emory Primate Center Biological Material Procurement program or through ongoing studies involving Rhesus macaques with approved IACUC. The human mobilized peripheral blood was obtained through discarded product post cell therapy obtained through Emory Clinic. The 4–5 mL bone marrow aspirate (BMA) specimen was diluted with 2% FBS-supplemented PBS at 1:1 ratio and filtered with 100-micron strainer. The strained BMA was directly layered over 15 mL lymphoprep (CAT #07851; STEMCELL TECHNOLOGIES, Tukwila, WA, USA) containing SepMate conical tube (CAT #85450; STEMCELL TECHNOLOGIES). Bone marrow scrap (BMS) was first resuspended in 25 mL 2% FBS-supplemented PBS and filtered using 100-micron strainer. The strained BMS was further diluted to 50 mL and split into two 25 mL sample each layered over 15 mL lymphoprep containing regular conical tube (CAT #430921, Corning, Corning, NY, USA). The BMA or BMS were centrifuged for 1500× *g* for 20 min. The buffy coat containing mononuclear cells was collected into a separate 50 mL falcon tube and washed with FBS-supplemented PBS by centrifugation at 300× *g* for 5 min. The cell pellet was treated with ACK lysis buffer to remove RBCs. The cell pellet was resuspended in 4 mL ACK buffer and incubated for 3–4 min at RT. After incubation, the ACK buffer/cell mixture was topped up with FBS-supplemented PBS and adjusted the volume to 30–50 mL depending upon the amount of initial BMS content and centrifuged at 300× *g* for 5 min. Cells were washed twice with FBS-supplemented PBS as before, and final pellet was resuspended in appropriate volume of buffer, counted, and used or stored. All steps and buffer used was kept chilled at all times.

### 2.8. Enrichment and Isolation of CD3^−^CD34^+^ HSPCs

Both Rhesus macaque and human CD34^+^ HSPCs were first enriched using Dynabeads CD34 isolation kit (CAT #11301D, Invitrogen) as per manufacturer’s protocol. Briefly, 40 × 10^6^ bone marrow mononuclear cells were mixed with 100 µL Dynabeads and incubated on ice for 30 min. Dynabead-treated cells were topped up with MACS buffer (CAT #20144, STEMCELL TECHNOLOGIES) to make final volume 2 mL. The tube containing bead-bound cells was placed under magnet for 2 min and unbound cells were discarded. This step was repeated one more time and the bead-bound cells were resuspended in 100 µL buffer. Next, Dynabeads were detached by adding 100 µL DETACHaBEAD, incubated for 45 min. To enhance detachment of beads, 3 mL MACS buffer was added to the bead-cell mixture and vortexed. The tube was placed under magnetic field for 2 min and supernatant with detached cells were collected in a separate tube. To ensure all cells were collected Dynabeads were further washed and subjected to magnetic field and supernatant was collected. The enriched CD34^+^ cells were washed with 10mL MACS buffer to remove excess DETACHaBEAD. The cells were either stored or subjected to FACS sort. For FACS, sort cells were stained with live/dead, CD34, and CD3. For generation of ATO CD3^−^CD34^+^ live cells were sorted using FACS.

### 2.9. Establishment of a Thymus-Independent Artificial Organoid

To culture RhATO, previously published protocol was adapted and modified [28]. Briefly, RhATO culture media was prepared by supplementing RPMI1640 (CAT #) with 3% B27 serum free media (CAT #17504001, Thermo Fisher Scientific), 1% Glutamax (CAT #35050061, Thermo Fisher Scientific), 1% penicillin–streptomycin (CAT #P4458, Thermo Fisher Scientific), 5 ng/mL stem cell factor (CAT #30007100UG, PeproTech, Rocky Hill, NJ, USA), 50 μM l-ascorbic acid (CAT # A8960, Sigma-Aldrich, Houston, TX, USA), 7.5 ng/mL recombinant IL-7 (CAT # 200-07-2UG, PeproTech), and 7.5 ng/mL FLT3 ligand (CAT #300-19-2UG, PeproTech). Appropriate number of six-well plates were filled with 1 mL culture media. Then, 0.4 μm Millicell Transwell insert (CAT #PICM0RG50, Merck) were placed into the culture media containing six-well plate. Next, Rh-DLL1 cell line was trypsinized, passed through 30-micron strainer to remove any cell aggregates, resuspended in culture media, and counted. After, 3000–7500 sorted CD3^−^CD34^+^ HSPCs were aggregated with rhDLL1 at 1:20 ratio and mixed by gently pipetting up and down few times. Cell aggregate was centrifuged at 300× *g* for 3 min. Supernatant was carefully aspirated, and cell pellet was resuspended in 5 μL culture media per RhATO. To establish RhATO, 5 μL of cell aggregate was carefully dropped on insert. Plate containing cell aggregates was incubated in humidified 37 °C incubator with 5% CO_2_. Media was replaced every 3–4 days and RhATO was cultured for 1 to 12 weeks depending upon requirement of experiment and number of T cells needed.

### 2.10. RhATO Phenotypic Analysis by Flow Cytometry

To establish the T cell developmental kinetics, RhATO were processed for phenotypic analysis at week 1, 2, and 4 of culture. Individual RhATO was processed by pipetting 1 mL FACS buffer onto the culture insert carrying the RhATO and gently disrupting the aggregate using the pipette tip. Broken aggregates were pipetted in and out a few times to ensure the release of lymphocytes into the solution. The lymphocyte rhDLL1 aggregate cell was filtered by passing through a 100-micron strainer, and surface immunostaining was performed. To analyze T cell developmental stages, cells were incubated with FACS buffer containing live/dead, CD45, CD34, CD38, CD3, CD8, CD4, CD7, TCR, CD56, CD20. The flow cytometry was performed using BD Symphony FACS analyzer and the data was analyzed using Flowjo v10.

### 2.11. T Cell Cytokine Assay

T cell cytokine assays were performed on 2 × 10^6^ cells each from peripheral blood mononuclear cells (PBMCs) and thymocytes per test. For RhATO derived T cells, CD3^+^ T cells were enriched from sixty RhATOs at 12 weeks of culture using Pan T cell isolation kit as per manufacturer’s protocol. Briefly, RhATO was processed as described above and cells were centrifuged at 300× *g* for 10 min. Supernatant was discarded and cell pellet was resuspended in 40 μL of MACS buffer (Cat #20144, STEMCELL TECHNOLOGIES). Then, 10 μL of Biotin-antibody cocktail was added to cells and incubated on ice for 10 min. Cells were washed with 2 mL MACS buffer and cell pellet was resuspended in 80 μL of MACS buffer followed by addition of 20 μL anti-Biotin microbeads. Mixture was incubated on ice for 15 min followed by washing with 2 mL buffer. Cell pellet was resuspended in 500 μL of buffer and subjected to magnetic separation. For magnetic separation, LS column was placed on magnetic stand (Cat #130042401, Miltenyi Biotec, Gaithersburg, MD, USA). Column was prepared by passing 3 mL MACS buffer followed by applying cell suspension. Flow through was collected in a sterile tube. Column was washed three times each with 3 mL MACS buffer and flow through was collected. All steps were performed under sterile conditions using chilled buffer. Collected T cell fraction was processed for stimulation with phorbol 12-myristate 13-acetate (PMA) and ionomycin (i), followed by intracellular cytokine staining as previously described [29]. Briefly, PBMCs, thymocytes, as well as RhATO derived T cells were seeded in 200 μL complete RPMI containing either PMA (80 ng/mL) plus i (1 μg/mL) or without PMAi. Mixture was incubated at 37 °C 5% CO^2^ for 2 h followed by addition of Glogi-stop (CAT #51-2092KZ, BD Biosciences) and Golgi-plug (CAT #512301KZ, BD Biosciences) and further incubation for 4 h. Intracellular cell cytokine staining: PMAi stimulated and unstimulated cells were washed with FACS buffer and incubated with 100 μL FACS buffer containing anti-CD3, IFNγ, TNFα, and IL-2 antibodies. Cell and antibody cocktail was incubated for 20 min on ice followed by washing with 500 FACS buffer twice. Upon second wash, supernatant was discarded and cell pellet was resuspended in 200 μL BD Cytofix/Cytoperm fixation and permeabilization solution (CAT # 554722, BD Biosciences) per test and incubated for 20 min on ice. Cells were washed twice with Perm wash buffer and further incubated with intracellular stain in 100 μL perm buffer containing anti-IFNγ, TNFα, and IL-2 antibodies. Cell antibody mix was incubated for 20 min on ice followed by two washes with 500 μL perm wash buffer (Cat #BDB554723; BD Biosciences) and a final wash with FACS buffer. Cells were resuspended in 200 μL FACS buffer and flow cytometry was performed.

### 2.12. Antibodies

Antibodies used in this study are as follows: CD3 (clone SP-34-2; BD Biosciences), CD45 (clone D058-1283 (RUO), BD Biosciences), CD8 (clone SK1; BD Bioscience), CD4 (clone L200; BD Biosciences), CD34 (clone 561, BioLegend, San Diego, CA, USA), CD38 (clone AT1), CD7 (clone M-T70, BD Biosciences), TNFα (MAb11; BD Biosciences), IL-2 (MQ1-17H12; BD Biosciences), IFNγ (B27; BD Biosciences), CD20 (clone 2H7, BioLegend), CD56 (clone NCAM162, BD Biosciences), and TCRαβ (clone R73, BioLegend).

## 3. Results

### 3.1. Generation and Characterization of MS5 Stromal Cells Expressing Rhesus macaque (Macaca mulatta) DLL1

**Figure 1:**
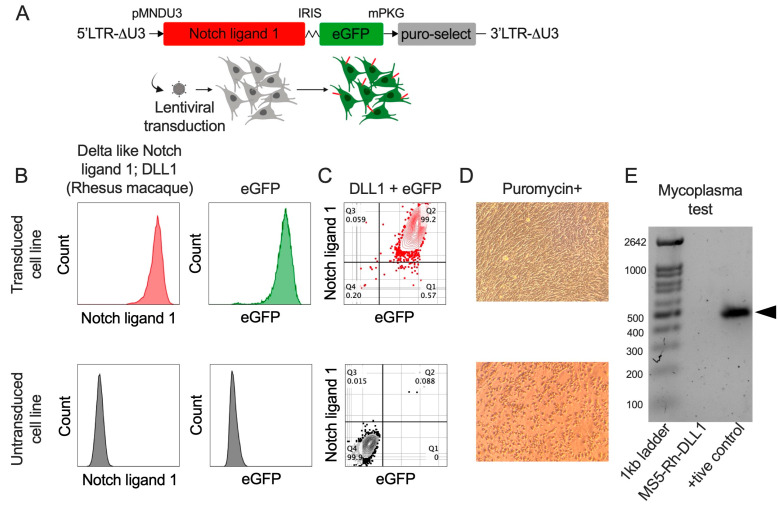
Characterization of a recombinant MS-5 stromal cell line expressing *Macaca mulatta* notch ligand 1 gene (**A**–**D**). (**A**) Cartoon showing lentiviral construct design with Rhesus macaque (*Macaca mulatta)* Notch ligand 1, eGFP reporter, and puromycin coding sequences. Arrows represent steps in transduction of MS-5 cell line (grey) with lentivirus coding indicated sequence to express RhDLL1 in modified MS-5 cell line (green). Created in BioRender. RAHMAN, S. (2025) https://BioRender.com/x8lpu3v. (**B**) Flow cytometry histogram plots showing positive surface expression of Notch ligand 1 (upper plot in red), eGFP (upper plot in green), in the transduced cell line and absence of their expressions in the non-transduced cell line (lower plots in gray). (**C**) Flow cytometry plots showing co-expression of DLL1 and eGFP (upper plot in red) and no expression (lower plot in gray) in the transduced and non-transduced cell lines, respectively. (**D**) Widefield image taken by iPhone mobile attachment at 5× showing viable transduced cell line (upper image) and dead non-transduced cells (lower image) cultured in the presence of 5 µg/mL puromycin. (**E**) Gel electrophoresis showing absence of mycoplasma in the newly established MS5-Rh-DLL1 cell line.

The murine bone marrow stromal cell line MS5 was genetically engineered to constitutively express Rhesus macaque Delta-like canonical Notch ligand 1 (RhDLL1). To achieve stable expression, a lentiviral construct encoding DLL1, an enhanced green fluorescent protein (eGFP) reporter, and a puromycin resistance selection cassette was designed for genomic integration (Figure 1A). Flow cytometric analysis confirmed efficient transduction, with more than 99% of MS5 cells co-expressing eGFP and Rh-DLL1 (Figure 1B,C). To eliminate non-transduced cells and enrich for DLL1-expressing populations, cultures were subjected to puromycin selection, resulting in exclusive survival of cells harboring the RhDLL1 cassette (Figure 1D). The MS5-RhDLL1 was free of any potential mycoplasma contamination (Figure 1E). The resulting MS5-RhDLL1 stromal cells were used to establish the Rhesus-artificial thymic organoid (RhATO) as described in the method section.

### 3.2. Three-Dimensional RhATO System Supported Robust T Cell Specification of Rhesus macaque HSPCs

**Figure 2:**
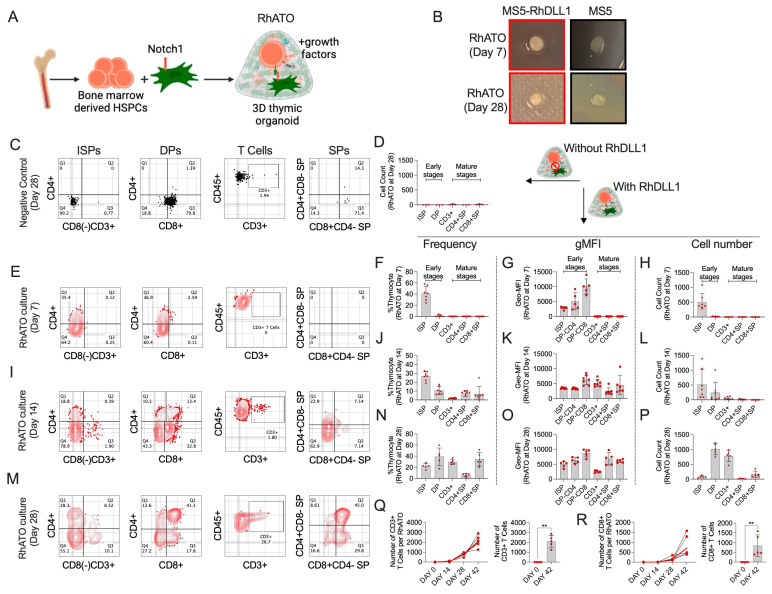
Establishment of Rhesus macaque-specific artificial thymic organoid (RhATO) system (**A**–**R**). (**A**) Cartoon showing RhATO establishment scheme. Created in BioRender. RAHMAN, S. (2025) https://BioRender.com/maircqp. (**B**) Images of 3D RhATOs established with MS5 expressing RhDLL1 (left panels) compared with 3D culture established with MS5 lacking RhDLL1 expression (right panels). The morphological features of 3D RhATOs are shown for week 1 (early) and week 4 (later) phases of T cell lineage differentiation post culture. (**C**–**P**) Early stages: CD4^+^CD3^−^ immature single-positive thymocytes (ISPs), CD4^+^CD8^+^ double-positive thymocytes (DPs); Mature stage: CD3^+^ T cell, CD3^+^CD4^+^, and CD3^+^CD8^+^ single-positive T cell subsets are shown. (**C**,**D**) Flow cytometry plots showing absence of T cell lineage differentiation ability of Rhesus bone marrow-derived HSPCs in 3D culture with MS5 lacking Rh-DLL1 expression (**C**), and bar graph showing total number of indicated cell generated at day 28 of culture (**D**). (**E**–**R**) Kinetics of T cell lineage differentiation stages generated in RhATO expressing RhDLL1. (**E**) Flow cytometry plots of thymocytes generated in RhATO at day 7 of culture establishment. (**F**–**H**) Bar graphs with individual data points showing frequency (**F**), geomean fluorescence intensity (G; gMFI), and number of cells (**H**) of early immature and late mature T cell differentiation stages generated in RhATO at day 7 of culture establishment. (**I**) Flow cytometry plots of thymocytes generated in RhATO at day 14. (**J**–**L**) Bar graphs with individual data points showing frequencies (**J**), gMFI (**K**), and number of cells (**L**) of early immature and late mature T cell differentiation stages generated in RhATO at day 14. (**M**) Flow cytometry plots of thymocytes generated in RhATO at day 28 of culture establishment. (**N**–**P**) Bar graphs with individual data points showing frequency (**N**), gMFI (**O**), and number of cells (**P**) of early immature and late mature T cell differentiation stages generated in RhATO at day 28 of culture establishment. (**Q**,**R**) Line graphs showing longitudinal track of number of T cells (**Q**) and CD8 T cells (**R**) generated per RhATO at day 0, 14 (week 2), 28 (week 4), and 42 (week 6) of culture establishment and bar graph showing direct comparison of cell numbers generated at day 42 (week 6) compared with day 0 of RhATO culture establishment. Experimental replicates: Total 19 RhATOs from three independent experiments were analyzed; N = 7 were analyzed at week 1, N = 7 were analyzed at week 2, and N = 5 were analyzed at weeks 4 and 6 of culture establishment. Statistical significance was calculated by Mann–Whitney U test. ** *p* < 0.01.

To evaluate functional significance of Rhesus DLL1 in 3D culture system, the RhATO was established by aggregating Rhesus bone marrow-derived CD34^+^CD3^−^ HSPCs with MS5 with and without RhDLL1 expression (Figure 2A,B), their morphological changes were recorded, and thymocyte generation was analyzed by flow cytometry. We examined the tightening of 3D aggregates over time as a sign of productive T cell differentiation, leading to their compaction and formation of dome-shaped 3D structures. At week 1 of culture, the RhATO demonstrated early signs of compaction compared with a 3D culture lacking RhDLL1 (Figure 2B, upper panel). By week 4, the 3D aggregates demonstrated remarkable compaction leading to the formation of a dome-shaped structure, whereas 3D aggregates lacking RhDLL1 failed to form a compact dome-shaped structure (Figure 2B, lower panel). Flow cytometry analysis of the cells generated in 3D aggregates lacking RhDLL1 failed to register a productive T cell lineage differentiation of the HSPCs, as indicated by the absence of hallmark early thymocyte population (CD4^+^CD3^−^ ISP, and CD4^+^CD8^+^ DP), and corresponding absence in the emergence of any CD3^+^ T cell subsets (Figure 2C,D).

Next, we analyzed T cell lineage differentiation of HSPCs in 3D culture expressing RhDLL1. The kinetics of the thymic developmental stages were analyzed by flow cytometry at weeks 1, 2, and 4 of the RhATO culture establishment (Figure 2E–P). At day 7 of the RhATO culture, ISPs dominated the population with frequency ~39% (range 24–58%), followed by DP population (~3%) (Figure 2E,F). The CD4 expression on ISP population showed ~2 times lower geomean fluorescence intensity (gMFI, ~2847) compared with their expression on DP (gMFI, ~5099), and ~3 times lower compared with the CD8 expression on DP population (gMFI, ~10105) (Figure 2G). The number of ISP cells ranged from ~300 to over 1000 (mean, ~503 cells) with few emerging DP cells (mean, ~16) (Figure 2H). By day 14, the frequency of ISPs decreased to ~27% (mean) compared with their frequency at day 7 (Figure 2J). The DP cells markedly expanded (mean frequency, 10%), accompanied by the emergence of CD3^+^ population (mean frequency, ~2%). At this stage, CD4^+^ (mean frequency, ~7%) and CD8^+^ (mean frequency, ~7%) single-positive cells also emerged (Figure 2I,J). Consistently, CD3 expression increased as indicated by gMFI of ~5187 (mean) (Figure 2K). Both the number of DP (mean 244) and CD3 (mean 53) increased at day 14 of culture (Figure 2L). By day 28, the frequency of ISP population in the RhATOs decreased by ~2-fold, their gMFI of CD4 expression increased by ~2-fold, and their number decreased by ~6-fold (Figure 2N–P). The frequency of DP population further increased to ~38% marking over a 40-fold increase compared with their frequency in day 7 RhATOs. The expression of CD8 (gMFI, 8738) remained higher compared with the expression of CD4 (gMFI, 6347) on DP population (Figure 2O). The mean number of ISP, DP, CD3, CD4, and CD8 cells were ~76, 1009, 783, 15, and 169, respectively, at day 28 of RhATO culture (Figure 2P). Next, we analyzed the number of CD3^+^ T cells and CD8^+^CD4^−^ single positive T cell subsets at day 42 of RhATO culture (Figure 2Q,R). The total number of T cells further increased to over 2000/RhATO by day 42 (week 6) of RhATO culture (Figure 2Q). Consistently, the number of CD8^+^ T cells/RhATO increased to over 700 cells by day 42 (Figure 2R). Taken together, these results demonstrated a robust RhDLL1 dependent T cell specification and developmental ontogeny of bone marrow-derived Rhesus macaque HSPCs.

### 3.3. RhATO-Generated Intermediate Thymocyte Populations Mirroring Bonafide Thymus

**Figure 3:**
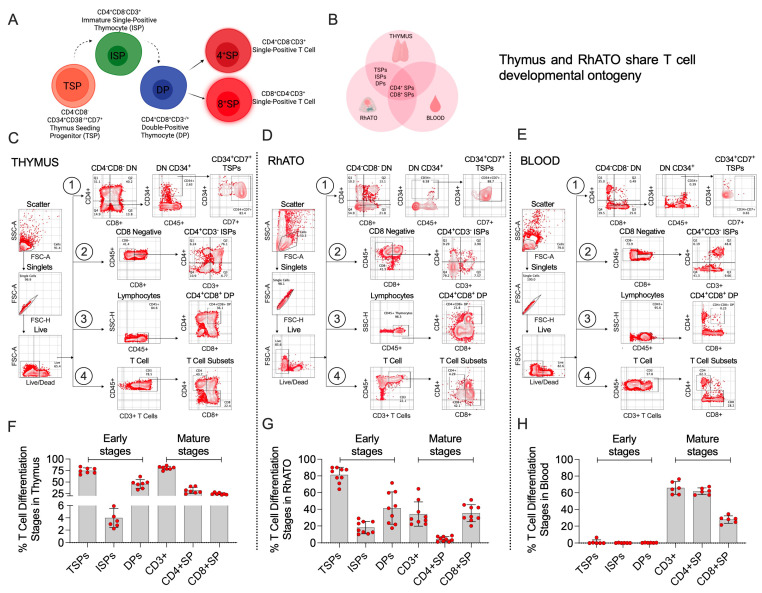
RhATO-generated thymocytes mirrored bona fide thymic intermediates (**A**–**H**). (**A**,**B**) Cartoon showing key developmental stages (**A**), and shared T cell developmental ontogeny between thymus, RhATO, and blood (**B**). Created in BioRender. RAHMAN, S. (2025) https://BioRender.com/56t84nx. (**C**–**E**) Flow cytometry plots showing gating strategy for CD4^−^CD8^−^ double-negative CD34^+^CD7^+^ thymus-seeding progenitors (TSPs; 1st row), CD4^+^CD3^−^ immature single-positive thymocytes (ISPs; 2nd row), CD4^+^CD8^+^ double-positive (DP) thymocyte (DPs; 3rd row), and CD3^+^, CD3^+^CD4^+^, CD3^+^CD8^+^ T cell subsets (4rth row) are indicated by numbers. The gating strategies are shown for thymus- (**A**), RhATO- (**B**), and blood-derived (**C**) T cells and their precursors. (F–H) Bar graphs showing individual data points representing frequencies of early thymocyte intermediates (TSPs, ISPs, and DPs) and mature T cell subsets (CD3^+^ T cells, CD3^+^CD4^+^, and CD3^+^CD8^+^ single-positive T cells). The data points are shown for cells analyzed for thymus- (**F**), RhATO- (**G**), and blood-derived (**H**) lymphocytes. Experimental replicates: Thymus and blood samples were obtained from Rhesus macaques (N = 6). For data in Figure 3B,E, two technical organoid replicates from four independent experiments (N = 8 organoids) were analyzed at day 28 of culture.

To establish that the 3D RhATO microenvironment recapitulates bona fide thymic function in supporting T cell lineage-biased differentiation of HSPCs, we analyzed multiple early thymic populations in Rhesus macaque thymus-, RhATO-, and blood-derived cells by flow cytometry (Figure 3A–H). To define early thymic progenitor phenotype, we examined thymus-seeding progenitors (TSPs), marked by the expression of CD7 in CD34^+^CD4^−^CD8^−^ double-negative progenitor population (Figure 3A–E). As expected, the thymus contained a markedly higher frequency of TSPs (mean, ~74.5%). Notably, the RhATO exhibited comparable TSP frequencies to those observed in the thymus (~81%; Figure 3D,G). The TSP population in the blood (mean, 1%) showed 75–80-fold lower frequency compared with their frequencies in the thymus or RhATO (Figure 3E,H).

Next, we compared frequencies of T cell-committed early thymocytes, including CD4^+^CD3^−^ immature single-positive (ISP) and CD4^+^CD8^+^ double-positive (DP) populations between the thymus, RhATO, and blood. In thymus, ISP population represented ~4% of the CD8 negative CD45^+^ fraction, and DP represented ~46% of the total CD45^+^ fraction of the thymocytes (Figure 3F). Similarly, in RhATO, ISP population represented ~18%, and DP represented ~41% of the thymocytes (Figure 3G). Whereas, in blood ISP and DP population represented ~0.3% and 0.43% of the lymphocytes, respectively (Figure 3H). Frequencies of CD3^+^ mature T cells in thymus, RhATO (day 28 culture), and blood showed ~80%, ~34%, and ~65% T cell population, respectively (Figure 3F–H). As expected, mature CD3^+^ T cells and their CD4^+^ and CD8^+^ single-positive subsets were detectable in thymus, RhATO, and blood but early thymocytes were predominantly observed in thymus and RhATO but not in blood. Taken together, these data demonstrate that the RhATO shares fundamental characteristics with the thymus in T cell developmental ontogeny.

### 3.4. The RhATO System Disfavored Non-T Cell-Biased Lineage Specification of HSPCs Akin to Thymus

**Figure 4:**
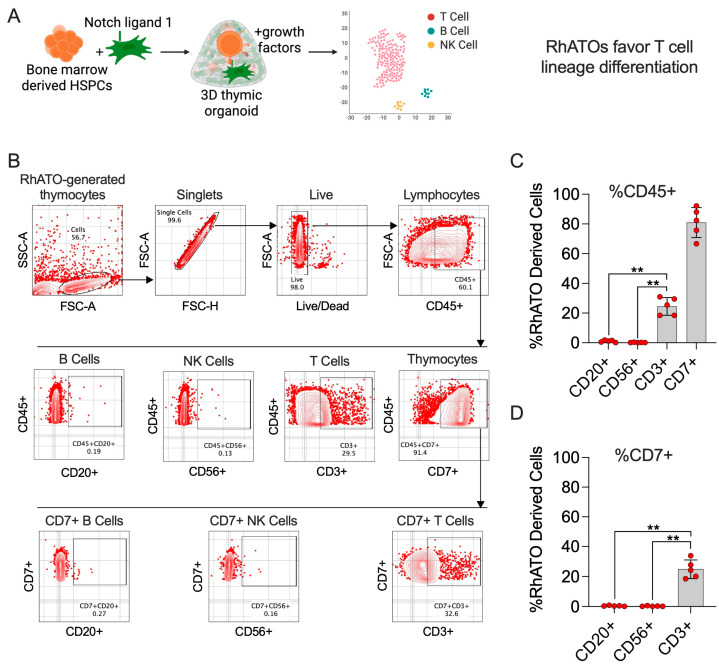
RhATO favored T cell-biased lineage differentiation of bone marrow-derived hematopoietic stem and progenitor cells (HSPCs) (**A**–**D**). (**A**) Cartoon showing RhATO-generated lineage analysis by flow cytometry. Created in BioRender. RAHMAN, S. (2025) https://BioRender.com/6gyooct. (**B**) Gating strategy showing flow cytometry analysis of distinct lymphocyte lineages (B, NK, and T cells) generated by RhATO system at week 4 of culture. (**C**) Bar graph with individual data points showing frequencies of B cells (CD20^+^), NK cells (CD56^+^), T cells (CD3^+^), and thymocytes (CD7^+^) as a frequency of total lymphocytes (CD45^+^). (**D**) Bar graph with individual data points showing frequency of B cells (CD20^+^), NK Cells (CD56^+^), and T cells (CD3^+^), as frequency of thymocyte (CD7^+^) population. Experimental replicates: A total of five organoids (N = 5) were analyzed; two technical organoid replicates from two independent experiments and one organoid from a third independent experiment were analyzed. Statistical significance was calculated by Mann–Whitney U test. ** *p* < 0.01.

To further validate the T cell-biased microenvironment of the RhATO, analogous to the thymus, we assessed the differentiation of HSPCs into common lymphoid lineages (T, NK, and B cells) at week four of culture by flow cytometry (Figure 4). RhATO cultures demonstrated a pronounced bias toward T cell (CD3^+^) differentiation, with CD3^+^ T cells comprising ~24% of total CD45^+^ lymphocytes, compared with markedly lower frequencies of B cells (~0.8%) and NK cells (~0.2%) (Figure 4B–D). Phenotypic profiling further revealed strong enrichment of CD7^+^ cells (~80%), a hallmark of thymocyte differentiation (Figure 4C). These CD7^+^ cells were predominantly biased toward the T cell lineage rather than B or NK cell differentiation (Figure 4D), reinforcing the T cell-specific nature of the RhATO niche.

**Figure 5:**
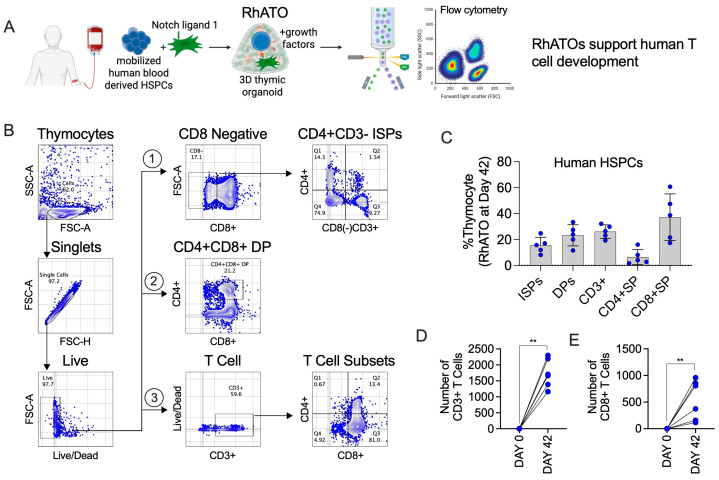
RhATO-supported T cell lineage differentiation of mobilized human blood-derived hematopoietic stem and progenitor cells (**A**–**E**). (**A**) Schematics of RhATO-mediated generation and analysis of human T cells. Created in BioRender. RAHMAN, S. (2025) https://BioRender.com/of6s47h. (**B**) Flow cytometry plots showing gating strategy for CD4^+^CD3^−^ immature single-positive thymocytes (ISPs; 1st row), CD4^+^CD8^+^ double-positive thymocyte (DPs; 2nd row), and CD3^+^, CD3^+^CD4^+^, CD3^+^CD8^+^ T cell subsets (3rd row) are indicated by numbers. (**C**) Bar graph showing individual data points representing frequencies of early TSPs, ISPs, DPs, and mature T cell subsets (CD3^+^ T cells, CD3^+^CD4^+^, and CD3^+^CD8^+^ single-positive T cells). (**D**,**E**) Bar graph representing individual data points showing number of T cells (**D**) and CD8 T cells (**E**) generated per RhATO at day 42 (week 6) compared with day 0 of culture establishment. The data points are shown for cells generated in RhATO using human derived HSPCs. Experimental replicates: five organoids (N = 5 organoids) from three independent experiments were analyzed at day 42 of culture. Statistical significance was calculated by Mann–Whitney U test. ** *p* ≤ 0.01.

To evaluate cross-species applicability, we aggregated human mobilized peripheral blood CD34^+^CD3^−^ HSPCs with MS5-RhDLL1 and analyzed thymopoiesis after six weeks (Figure 5A–E). Human HSPCs generated intermediates and mature T cell subsets, including ISPs (15%), DPs (22%), and CD3^+^ T cells (26%), with CD4^+^ (~5%) and CD8^+^ (~33%) single-positive populations closely resembling outcomes observed with rhesus-derived HSPCs. Next, we analyzed the number of CD3^+^ T cells and CD8^+^CD4^−^ single-positive T cell subsets at day 42 (week 6) of RhATO culture (Figure 5D,E). The total number of T cells generated increased to ~1734 per RhATO (Figure 5D), whereas CD8^+^ single-positive cells increased to ~544 per RhATO (Figure 5E) compared with their numbers on day 0 of culture.

Together, these findings demonstrate that RhATO faithfully recapitulates thymic cues required for T cell lineage specification from rhesus CD34^+^ HSPCs while also supporting T cell development from human HSPCs, enabling accurate ex vivo modeling of T cell developmental ontogeny.

### 3.5. RhATO-Generated Mature T Cells Shared Features of Recent Thymic Emigrants

**Figure 6:**
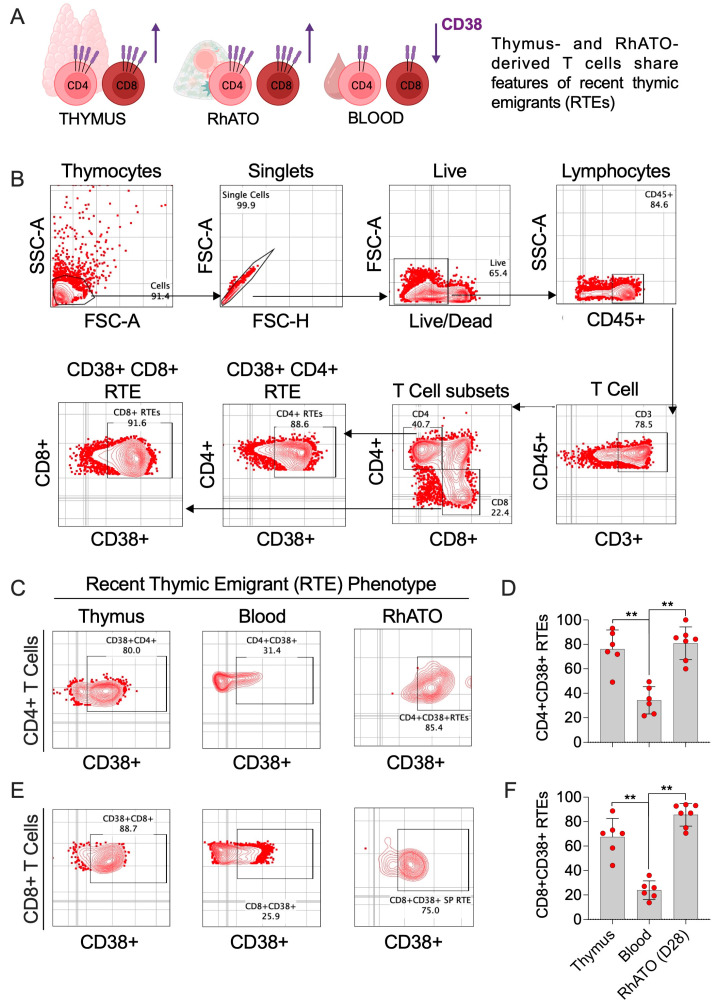
RhATO-generated T cells mirrored recent thymic emigrant (RTE) phenotypic characteristics known in bona fide thymus-generated mature T cells (**A**–**E**). (**A**) Cartoon showing CD38 T cell expression pattern on T cells between thymus-, RhATO-, and blood-derived cells as a marker for RTEs in Rhesus macaques. Created in BioRender. RAHMAN, S. (2025) https://BioRender.com/db5wcx7. (**B**) Flow cytometry plots showing gating strategy for CD38 expressing T cell subsets in thymus. (**C**) Flow cytometry plots showing CD38 expressing CD4 T cell subsets in thymus (as a positive control), blood (as a negative control), and RhATO-derived cells. (**D**) Bar graph showing individual data points representing frequencies of CD38 expressing CD4^+^ T cell subset. (**E**) Flow cytometry plots showing CD38 expressing CD8 T cell subsets in thymus-, blood-, and RhATO-derived cells. (**F**) Bar graph showing individual data points representing frequencies of CD38 expressing CD8^+^ T cell subset. The data points are shown for cells generated in RhATO at day 28 of culture using Rhesus macaque-derived HSPCs. The frequencies of RTEs generated in RhATO is compared with the corresponding RTE population derived from thymus and blood of the RM. Experimental replicates: Thymus and blood samples were obtained from six Rhesus macaques (N = 6). For data in Figure 3C–E, six organoids (N = 6) from three independent experiments were analyzed for RTEs. Statistical significance was calculated by Mann–Whitney U test. ** *p* ≤ 0.01.

Here, we analyzed the features of recent thymic emigrants (RTEs) on T cell subsets generated in RhATO compared with T cell subsets derived from a bona fide thymus as well as blood from Rhesus macaques (Figure 6A). We analyzed the proportion of T cell subsets expressing CD38 as a phenotypic marker of RTEs on CD4^+^ and CD8^+^ T cell subsets (Figure 6B–F). The proportion of CD38 expression in the thymus-derived CD4^+^ T cell subset showed a frequency of ~76%, in blood this frequency was ~34%, and in RhATO this frequency was ~81% (Figure 6C,D). Both thymus- and RhATO-derived CD4 T cells showed over 2-fold increase in the proportion of cells expressing the CD38 marker. Next, we analyzed the proportion of CD8 T cell subset expressing an RTE marker. The proportion of CD38 expression on the thymus-derived CD8^+^ T cell subset showed a frequency of ~68%, in blood this frequency was ~24%, and in RhATO this frequency was ~86% (Figure 6E,F). Both thymus- and RhATO-derived CD8 T cells showed ~3-fold increase in the proportion of cells expressing the CD38 marker compared with CD8 T cells in blood. These results demonstrate that the RhATO generated terminally differentiated T cell subsets showing phenotypic features of RTEs.

### 3.6. RhATO Supported Efficient TCR-Selected T Cell Development with Functional Potential

**Figure 7:**
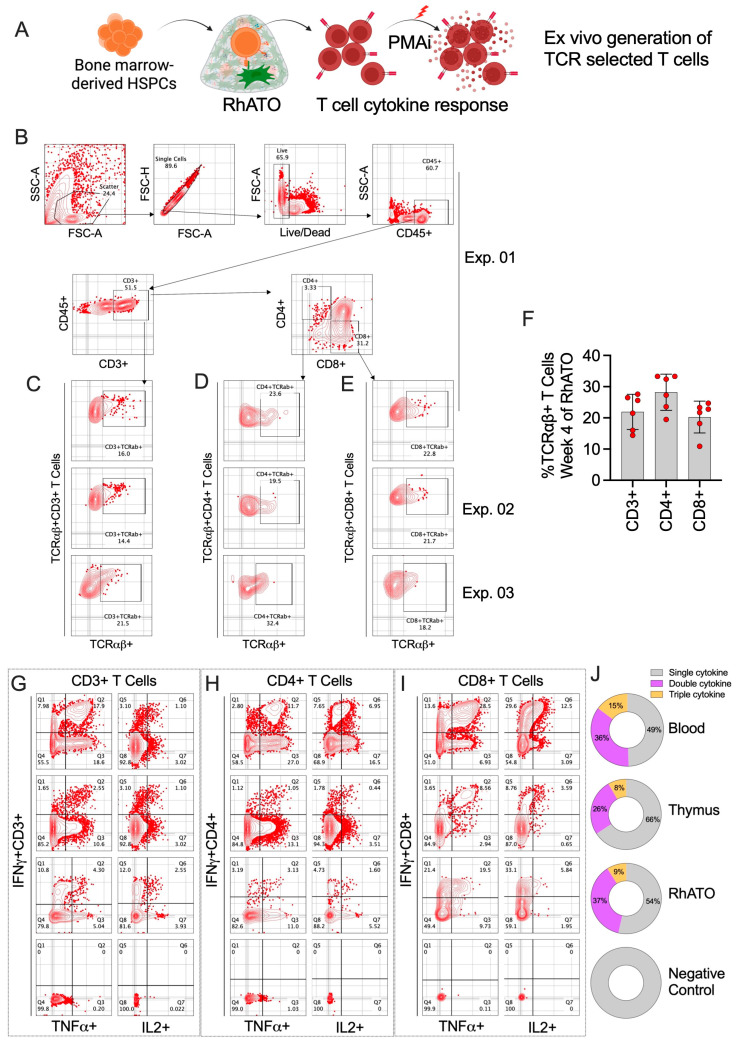
RhATO demonstrated efficient generation of TCR-selected T cell subsets from Rhesus macaque bone marrow-derived HSPCs within four weeks of culture (**A**–**J**). (**A**) Schematics showing phenotypic and functional characterization of TCR-selected T cells generated in RhATO. Created in BioRender. RAHMAN, S. (2025) https://BioRender.com/giphpbd. (**B**–**J**) Flow cytometry plots showing gating strategy (**B**) for TCRαβ selected CD3^+^ T cells (**C**), CD3^+^CD4^+^ (**D**), and CD3^+^CD8^+^ (**E**) single-positive T cell subsets. The flow plots for TCRαβ-selected T cell subsets are shown from three independent experiments (Exp1, Exp2, and Exp3). (**F**) Bar graph showing individual data points representing frequencies of TCRαβ-expressing CD3^+^ T cells, CD4^+^, and CD8^+^ single-positive T cell subsets. (**G**–**I**) Flow cytometry plots showing indicated cytokine expression by CD3^+^ T cells (**G**), CD3^+^CD4^+^ (**H**), and CD3^+^CD8^+^ (**I**) T cell subsets are shown for cells derived from peripheral blood-derived lymphocytes, thymus-derived thymocytes, and RhATO-derived T cells. Cells stimulated with phorbol 12-myristate-13-acetate (PMA), and ionomycin (upper three panels), or no stimulation (lower panel) are shown. RhATO (N = 6) generated and enriched T cells expressing IFNγ^+^, TNFα^+^, and IL2^+^ cytokines. (**I**) Boolean analysis of cytokine co-expression is shown for PMAi-stimulated (upper panel) and unstimulated (lower panel) T cells. Single cytokine = IFNγ^+^, TNFα^+^, and IL2^+^; Double cytokine = co-expression of any following combinations: IFNγ^+^/TNFα^+^, IFNγ^+^/IL2^+^, TNFα^+^/IL2^+^; and Triple cytokine = co-expression of IFNγ^+^/TNFα^+^/IL2^+^ cytokines. A total of sixty RhATOs were pooled for the cytokine analysis.

To evaluate whether T cells generated in the RhATO system undergo productive TCR rearrangement, we first assessed expression of TCRαβ, the predominant TCR subtype on the RhATO-generated T cells and their subsets (Figure 7A–J). At four weeks of culture, ~21% of CD3^+^ T cells expressed TCRαβ (Figure 7C,F), with both CD4^+^ and CD8^+^ single-positive subsets showing ~27% (Figure 7D,F), and ~20% (Figure 7E,F) TCRαβ^+^ single-positive subsets, respectively. Next, we examined the functional capacity of RhATO-derived T cells by quantifying cytokine responses following stimulation and compared the responses with T cells in blood and thymus (Figure 7G–J). CD3^+^ T cells stimulated with PMA/ionomycin (PMAi) exhibited robust induction of IFN-γ, TNF-α, and IL-2 in all three compartments (blood, thymus, and RhATO) compared to unstimulated controls (Figure 7G–I). Both CD4 (Figure 7H) and CD8 (Figure 7I) T cell subsets also demonstrated cytokine expression upon stimulation. Boolean analysis revealed that 49% (in blood), 66% (in thymus), and 54% (in RhATO) of T cells produced at least one cytokine (single cytokine responders. In addition, 36% (in blood), 26% (in thymus), and 37% (in RhATO) of the T cells co-expressed two cytokines. Finally, 15% (in blood), 8% (in thymus), and 9% (in RhATO) of T cells co-expressed all three cytokines, indicating a polyfunctional response profile (Figure 7J). Collectively, these results demonstrated that RhATO-generated T cells acquired productive TCRαβ expression and developed polyfunctional effector capacity, closely mirroring thymus-derived T cell function and confirming the fidelity of the RhATO platform for developing functional TCR selected T cells.

## 4. Discussion

In this study, we describe the development and characterization of a Rhesus macaque-specific artificial thymic organoid (RhATO) system that supports efficient ex vivo T cell lineage-biased differentiation of bone marrow-derived hematopoietic stem and progenitor cells (HSPCs). The RhATO recapitulated key stages of thymopoiesis, including the emergence of CD4^−^CD8^−^ double-negative CD7^+^CD34^+^ progenitors resembling thymus-seeding progenitors (TSPs), progression to CD4^+^CD3^−^ immature single-positive (ISP) and CD4^+^CD8^+^ double-positive (DP) early thymocytes, and maturation into CD4^+^ and CD8^+^ single-positive CD3^+^ T cells expressing functional TCRs. Importantly, these T cells exhibited polyfunctional cytokine responses, indicating acquisition of immunological competence. To our knowledge, this is the first nonhuman primate (NHP)-specific, primary thymus-tissue-free system that faithfully reproduces canonical thymic development.

Earlier ex vivo T cell generation systems relied on murine stromal lines expressing DLL1 or DLL4 (e.g., OP9-DLL1, MS5-DLL1), which effectively directed HSPCs toward T cell lineage differentiation [6,17,18,19,20,23,25,26,30]. However, these approaches frequently yielded suboptimal CD8^+^ T cell output and required prolonged culture periods. Advances in 3D organoid culture have underscored the importance of spatial organization for efficient T cell selection and maturation [27,28,31]. Our findings extend this concept to the NHP setting by demonstrating that a rhesus-specific DLL1-expressing MS5 cell line can drive robust T cell development in a 3D configuration. Compared with human DLL1-based ATOs, the RhATO achieved differentiation of higher frequencies of mature CD4^+^ (~5%) and CD8^+^ (~32%) T cells as well as robust TCR-selected T cell development within four weeks of culture and exhibited more rapid organoid compaction (7 days). The use of species-specific, puromycin-selected, MS5-RhDLL1 cells, which ensured specific and high DLL1 expression, may underlie this enhanced and accelerated T cell lineage specification of Rhesus macaque HSPCs. Moreover, the RhATO also supported human HSPC-derived T cell development, underscoring its cross-species utility. In general, we observed a markedly higher generation of CD8^+^ T cells compared to CD4^+^ T cells within the RhATO system, irrespective of the HSPC source (nonhuman primate or human). This observation aligns with previous reports [27,28] and is likely attributable to the limited presence of MHC class II-expressing cells required for CD4^+^ T cell selection, which are relatively scarce in ATOs, in contrast to the more ubiquitous expression of MHC class I molecules on various cell types, including HSPCs.

Quantitative analysis revealed an average of ~2000 (range 1292–2875) CD3^+^ T cells per RhATO by day 42 of culture, including ~700 (range 391–1576) CD8^+^ T cells. While these yields are comparable to those reported in prior human ATO systems using bone marrow-derived HSPCs [28], a greater expansion of CD8^+^ T cells has been documented with cord blood-derived HSPCs and longer cultures. Thus, although T cell developmental ontogeny remains consistent and can be reliably investigated by day 28 of RhATO culture, the magnitude of T cell output is influenced by the source of HSPCs and length of culture. Selection of an optimal progenitor source should therefore align with experimental goals, particularly when large-scale T cell production is desired.

Although NHPs have been instrumental in investigating thymic output under pathogenic condition such as simian immunodeficiency virus infection [32], the impact of pathogenic inflammation on the T cell potential of bone marrow-derived progenitors is difficult to track in vivo. A non-animal technology, such as RhATO, has potential to facilitate these investigations considering it demonstrates T cell developmental ontogeny observed in the thymus. Phenotypic analyses confirmed that RhATO cultures generate hallmark thymocyte intermediates, including CD34^+^CD38^−^CD7^+^ TSP-like cells, CD4^+^CD3^−^ ISPs, and CD4^+^CD8^+^ DPs, culminating in mature CD4^+^ and CD8^+^ single positive CD3^+^ T cells. The emergence of CD38^+^ single-positive subsets, analogous to recent thymic emigrants, further supported the fidelity of this platform to physiological thymopoiesis. The detection of TCRαβ-expressing cells capable of producing IFN-γ, TNF-α, and IL-2 highlights the functional maturation achieved within this system. Earlier attempts to generate T cells from NHP HSPCs employed human or porcine thymic stromal cells [11,12,33], or murine OP9-DLL1 co-cultures [21]. While informative, these systems were limited by dependence on primary thymic tissue or incomplete recapitulation of T cell development, particularly the CD8^+^ lineage. The RhATO system overcomes these barriers by providing a renewable, standardized platform that eliminates the need for primary thymic stroma while enabling full developmental progression, including functional TCR acquisition.

In conclusion, we have established and characterized a Rhesus macaque-specific, primary thymus-tissue-free artificial organoid platform that supports complete T cell differentiation from bone marrow-derived hematopoietic stem and progenitor cells (HSPCs). This organoid system recapitulates canonical thymic development and generates functionally competent T cells. It represents a robust and versatile model to investigate T cell developmental ontogeny, delineate the impact of pathogenic inflammation, host factors, and viral determinants on the T cell differentiation potential of progenitors, and facilitate the generation of T cell receptor (TCR)-selected T cells for therapeutic applications.

## Data Availability

The raw data were generated at the Emory University and will be available upon request from the corresponding author.
